# Examining Controlling Styles of Significant Others and Their Implications for Motivation, Boredom and Burnout in Young Swimmers

**DOI:** 10.3390/ijerph18115828

**Published:** 2021-05-28

**Authors:** Octavio Alvarez, Lluis Tormo-Barahona, Isabel Castillo, Juan Antonio Moreno-Murcia

**Affiliations:** 1 Department of Social Psychology, University of Valencia, 46010 Valencia, Spain; octavio.alvarez@uv.es; 2 Sports Research Centre, Department of Sports Sciences, University Miguel Hernández, 03202 Elche, Spain; lluiscoach@gmail.com (L.T.-B.); j.moreno@umh.es (J.A.M.-M.)

**Keywords:** controlling style, coach, parents, peers, motivation, boredom, burnout

## Abstract

The aim of the study was to examine the controlling style in two contexts of social influence: the team (i.e., coach and teammates) and the family (i.e., father and mother), as well as the mediational role of motivation (autonomous, controlled, and amotivation) and its relationship with boredom and burnout in young swimmers. To this end, 267 swimmers (140 girls and 127 boys) between 12 and 18 years of age (M = 14.26; SD = 1.61) were assessed. The results showed that in the team context, coaches’ controlling style directly promoted controlled motivation and boredom in their swimmers, and indirectly influenced burnout through the mediating role of swimmers’ controlled motivation. Teammates’ controlling style was directly associated with controlled motivation, amotivation, and burnout, and indirectly associated with boredom and burnout through the mediating role of amotivation. Regarding the family context, the father’s controlling style showed direct associations with controlled motivation and burnout, and indirect associations with boredom through the mediating role of swimmers’ controlled motivation. Finally, the associations of the mother’s controlling style with all the variables studied were neutralized by the father’s controlling interpersonal style. This study emphasizes the differentiating role of significant others when displaying controlling styles, and it confirms that the controlling style has a significant relationship with maladaptive sport practice experiences.

## 1. Introduction

The factors that influence people’s experiences in the physical and sport domains and the quality of their experiences have been important targets of sport psychology research, due to the implications of sport participation for health and psychosocial development [1,2]. Despite the importance of being physically active, children and adolescents do not always receive the potential benefits of practicing sport, perhaps due to the psychological pressures athletes sometimes perceive from their significant others (e.g., coach or parental pressure). Among the factors that influence sport participation, one of the aims has been to explore how the psychological environment created by significant others (i.e., coaches, parents, and teammates) may foster adaptive and maladaptive motivational outcomes in sport participants [2,3,4].

### 1.1. Cognitive Evaluation Theory

The cognitive evaluation theory (CET; [5]), a mini-theory of the self-determination framework (SDT; [6,7]), proposes that social contexts influence the quality of a person’s motivation, as well as experiences of well-being and ill-being. Two interpersonal styles in relationships with significant others (i.e., coaches, parents, and teammates) have been considered in the literature, the autonomy supportive style and controlling features of the interpersonal style. The autonomy-supportive style encourages the person’s choice, self-regulation, and expression of emotions, opinions, and views, providing positive and informative feedback [7]. It is assumed that this interactive style will lead to positive outcomes (e.g., facilitate autonomous motivation, greater engagement, foster optimal psychological functioning, and promote well-being). In contrast, the controlling interpersonal style is characterized by the use of motivational strategies based on extrinsic rewards, negative feedback, control, and pressure. Thus, the one who is using this style tries to impose a specific and preconceived way of thinking and behaving, which in turn promote athletes’ ill-being [7,8]. Additionally, it has been suggested that with this controlling style, athletes are likely to exhibit negative affective, cognitive, and behavioral responses [6,7]. The consequences of an autonomy-supportive style have been widely studied in the literature (for a systematic review, see [9]), especially with respect to the coach and the physical education teacher. However, limited attention has been paid to other social groups (i.e., parents and teammates) and the consequences of using an interpersonal controlling style [10].

The present research is based on the SDT and aims to predict two indicators of ill-being in a sample of young swimmers, namely swimmers’ feelings of boredom during sport practice and reported burnout, considering the controlling interpersonal style of significant others (i.e., coach, teammates, and parents) and forms of motivation as antecedents.

### 1.2. Organismic Integration Theory

The SDT, throughout the organismic integration theory (OIT; [11]), links subjects’ tendency to internalize cultural and social regulations and how the social context can enhance or reduce this internalization process. This mini-theory proposes a continuum from controlled to autonomous motivation [7], distinguishing between intrinsic, extrinsic, and amotivation.

Additionally, the OIT distinguishes four subtypes of extrinsic motivation that define the degree to which extrinsically motivated behavior reflects the subject’s values and interests. Thus, from lower to higher motivational quality, a distinction is made between external, introjected, identified, and integrated regulation. External regulation is controlled through environmental contingencies (i.e., reward or punishment). When behavior is regulated through introjection, people perform the behavior by internalizing the external pressure, producing tension, conflict, and guilt when they do not perform the behavior. External and introjection regulations represent controlled forms of motivation. Identified regulation and integrated regulation are the two most self-determined extrinsic regulations [7]. They include understanding and accepting the values that encompass the behavior in the case of identified regulation and integrating the behavior with one’s own values and beliefs in the case of integrated regulation. Integrated regulation and intrinsic motivation are considered the most self-determined forms of motivation. The only difference is that in intrinsic motivation, the person is motivated by the behavior itself, whereas in integrated regulation, motivation comes from the achievement of goals related to the behavior, but not from the behavior itself. Thus, to the extent that behavior is more self-determined, it will have greater organismic support for action. Identified, integrated, and intrinsic motivation represent autonomous forms of motivation. Finally, amotivation describes passivity and a lack of purpose for action, with regard to a given behavior. It represents the lowest degree of motivation quality and is, in fact, the lack of it [7]. Amotivation has been identified as a precursor of maladaptive experiences in athletic participation, such as burnout (e.g., [12,13,14], boredom [15], and dropping out [16].

### 1.3. Interpersonal Styles and Implications for Athletes’ Motivation and Ill-Being

Both interpersonal styles (i.e., autonomy support and controlling style) in relationships with significant others (i.e., social influence agents such as coaches, parents, and teammates) have been considered antecedents of types of motivation in the literature (e.g., [7,17]. On the one hand, autonomy-supportive styles promote the internalization of athletes’ learning motivation, thus activating their autonomous motivation [7]. On the other hand, controlling styles by these social influence agents promote poor quality motivation and externalizing regulations (i.e., controlled forms of motivation), and they may even promote amotivation through mechanisms of coercion or seduction by means of punishments or rewards (also known as carrot and stick), disregarding the individual’s emotions and beliefs. The low-quality motivation fostered by the controlling interpersonal style is the reason the literature has widely identified its consequences for people’s health and engagement or for dropping out of the activity (e.g., [16,17,18]). For example, Pelletier et al. [17], in a classic longitudinal study in a sample of elite and non-elite swimmers, found that the coach’s controlling style promoted controlling motivational regulations, and, in turn, these regulations promoted swimmers’ attrition and dropout. One of the most commonly used variables to assess ill-being is athlete burnout perceptions. Burnout is a syndrome characterized by emotional and physical exhaustion in the athlete that leads to a loss of motivation and usually progresses to feelings of failure, loss of self-fulfillment, and devaluation of participation in sport [19,20]. Research has suggested that burnout is associated with many negative outcomes, including premature sport dropout [21].

Significant others have been identified as precursors of athletes’ burnout experiences [22], and the role of interpersonal styles of coaches (e.g., [1,23]), parents (e.g., [3,10]), and teammates (e.g., [24]) have been examined as predictors of burnout. Previous literature has pointed out that limited studies have examined the parent’s role in their adolescent children’s burnout [10]. Moreover, as far as we know, studies focused on other social groups, such as the impact of teammates’ behavior on athlete burnout, are even more scarce. Smith et al. [24] studied the role of motivational climates among teammates and their implications for burnout in adolescent athletes, showing the positive effect of intra-team conflict on burnout in individual athletes. Kiuru et al. [25] studied the role of peers in the development of burnout in the school context, suggesting that, in adolescence, the most important life issues are discussed with peers [26]. Thus, through interactions among peers, emotions and attitudes towards these important issues are shared and transmitted, producing processes of influence, for example, on the burnout levels of their peers [25]. Chan et al. [2], in a sample of swimmers, assessed the impact of the social influence of coaches, parents, and teammates on motivational patterns, and they found that the social influence of teammates was greater in adolescent athletes compared to athletes in other age groups. The authors concluded that age moderated the impact of social influence from significant others on young athletes’ sport experiences.

In this vein, motivational regulations have also been identified as predictors of burnout (e.g., [4,27,28,29,30,31]). In their systematic review of the burnout-in-sport-literature, Goodger et al. [32] conclude that in 100% of the published studies assessed, intrinsic motivation was negatively associated with burnout, and amotivation was positively associated with burnout, whereas extrinsic or controlled motivation was less clear but generally ranged from no association to small, negative associations. After this review, Balaguer and colleagues’ [28] results revealed that autonomous motivation negatively predicted burnout, whereas controlled motivation and amotivation were positively linked to burnout. A more recent study by Atienza et al. [4] with vocational dancers showed that autonomous motivation was negatively associated with burnout, and amotivation was positively associated with it, whereas controlled motivation was not linked to burnout. Other studies (e.g., [33,34]) have reported that perception of a controlling interpersonal coaching style had significant predictive power on thwarting each of the basic psychological needs (i.e., autonomy, competence, and relatedness), and these thwarted needs were positively associated with burnout.

One of the reasons offered to explain why youth drop out of sports is a lack of enjoyment, that is, being bored [35]. Boredom is defined as an aversive state where the individual is unable to successfully engage the internal or external attention required to participate in a satisfying activity. The subject is aware of this situation and attributes this aversive state to the environment [36]. Boredom has been linked to repetitive tasks in sports (e.g., [37]), and it is suggested that it undermines athletes’ motivation (e.g., [38]), decreasing the intention to continue to be physically active [39].

Competitive swimming demands great dedication from participants, due to the number of hours of training and high pressure during competitions. Although swimming is often categorized as an individual sport, swimmers do their training and even competitions as a team, and so they experience social interactions with their teammates, coaches, and parents [2]. Moreover, previous literature underlined the importance of significant others in the attrition process in young swimmers (e.g., [40]). Some of the suggested causes of having a bad experience in competitive swimming and dropping out are conflicts of interest, problems with the coach and significant others (parents, friends, etc.), boredom, and excessive pressure (e.g., [40]).

As Pacewicz and colleagues [41] pointed out, within the social context of sport, the effect of social interactions may vary according to the specific type of relationship (i.e., swimmer–coach, swimmer–swimmer, swimmer–parent). That is, the pressure applied by the coach may differ from the pressure from a teammate or a parent. Therefore, it would be interesting to understand the mechanisms operating in the two main social contexts of the adolescent swimmer: the sport context (i.e., coach and teammates) and the family context (i.e., parents or equivalent social agents), in relation to the likelihood of developing burnout syndrome and feeling bored at formative ages.

### 1.4. Aim of This Study

Although the influence of significant others on youth sport participants has received considerable attention from researchers in the sport domain (e.g., [42]), a limited number of studies have considered the four significant others mentioned above (e.g., [43,44]). In particular, there is a lack of studies that explore the role of teammates’ controlling interpersonal style in the development of burnout in adolescent athletes.

Consequently, the aim of this correlational study with a non-experimental, quantitative, cross-sectional design, was to explore through path analysis, in two different contexts of social interaction (i.e., team and family), the relationships between the controlling style used by significant others (i.e., coach, teammates, and parents), motivational regulations, the development of burnout syndrome, and boredom, in a sample of young swimmers in formative ages (between 12 and 18 years old). The following hypotheses will be tested:

**Hypotheses 1** **(H1).** *Controlling styles of significant others will be positively associated with controlled motivation and amotivation, and negatively associated with autonomous motivation.*

**Hypotheses 2** **(H2).** *Autonomous motivation will be negatively associated with burnout and boredom, and controlled motivation and amotivation will be positively associated with burnout and boredom.*

**Hypotheses 3** **(H3).** *Forms of motivation will act as mediators in the relationship between controlling styles of significant others and boredom, as well as in the relationship between controlling styles of significant others and burnout.*

## 2. Materials and Methods

### 2.1. Participants

A convenience sample consisted of 267 young swimmers (127 boys and 140 girls) belonging to three categories: alevin (*n* = 90; 12–13 years), infantil (*n* = 117; 14–15 years), and junior (*n* = 60; 16–18 years), aged 12 to 18 years (M = 14.26; SD = 1.61). Participants represented 12 different swimming clubs registered in the Valencian Community Swimming Federation (Spain) and gave their consent to participate in this study. They had been swimming for an average of 7.38 years (SD = 2.96) and had 8.58 (SD = 2.36) sessions of swimming per week. They were competing (from the lowest to the highest competitive level) at the regional level (*n* = 38), the autonomous community level (*n* = 98), and the national level (*n* = 117). The other swimmers (*n* = 14) did not indicate their level of competition. The inclusion criteria were being federated swimmer, being between 12 and 18 years old, and having expressed written consent to participate. Those who did not meet these criteria were excluded.

### 2.2. Measures

Coaches’, father’s, mother’s, and teammates’ controlling style. To assess the swimmer’s perception of the controlling style of the coach, father, mother, and teammates, the Spanish version of the Controlling Style Scale was adapted to this study [45]. This unidimensional scale is composed of nine items (e.g., “Talks continuously and does not allow me to make contributions at training/at home”) starting with the statement “During training and/or at home, your coach, your father, your mother, your teammates...”. Responses are collected on a Likert scale ranging from 1 (definitely no) to 5 (definitely yes). Previous research has confirmed the reliability of this scale in the sport context (e.g., [46]) and in the physical education domain (e.g., [45]).

Motivation. Swimming motivation was assessed with the Spanish version [47] of the Behavioral Regulation in Sport Questionnaire [48] adapted for swimmers. On this 24-item scale, divided into six subscales, swimmers are asked to think about why they participate in swimming. The scale begins with the sentence “I swim...”. Examples of items on each subscale are as follows: “Because I enjoy it” (Intrinsic motivation), “Because it is a part of who I am” (Integrated regulation), “Because I value the benefits of swimming” (Identified regulation). “Because I would feel guilty if I quit” (Introjected regulation). “Because if I don’t, other people will not be pleased with me” (External regulation), “But I wonder why I continue” (Amotivation). Responses are rated on a Likert-type scale ranging from 1 (not true at all) to 7 (completely true). Previous research with athletes has confirmed the reliability of the instrument (e.g., [47,48]). Consistent with the indications on the SDT [49] website, items from the intrinsic motivation, integrated, and identified regulation scales were combined to create the Autonomous motivation variable, and items from the introjected and external regulation scales were combined to create the Controlled motivation variable. That is, the latent variables are created by calculating the mean of all the items that make up the different scales.

Boredom. To assess the perception of boredom, we used the Spanish subscale [50] of the Sport Satisfaction Instrument [51] adapted to swimmers for this study. Swimmers are asked to indicate their degree of agreement with the two items that reflect boredom criteria (e.g., When I swim, I usually wish the swimming training or competition would end quickly). Responses are rated on a 5-point Likert scale ranging from 1 (strongly disagree) to 5 (strongly agree). Previous research has confirmed the reliability of this subscale (e.g., [38,50]).

Burnout. We used the Spanish version [28] of the Athlete Burnout Questionnaire [52] modified for the swimmer population. This scale has 15 items divided into three dimensions (reduced sense of accomplishment, swimming devaluation, and emotional and physical exhaustion). The scale begins with the sentence “How do you currently feel about your swimming participation?”, and the responses are rated on a 5-point-scale ranging from 1 (never) to 5 (always). For this study, a composite score reflecting global burnout was used. An example item is “I feel overly tired from my swimming participation”. Previous research studies in the sport domain have confirmed this instrument’s validity and reliability (e.g., [14,53]).

### 2.3. Procedure

The study was conducted according to the guidelines of the Declaration of Helsinki, and ethical approval was obtained from the Ethics Committee of the University Miguel Hernández of Elche (Alicante, Spain) (Code: 210517113557).

We contacted the directors of the swimming clubs to inform them about the goal of the study and request their collaboration. In the clubs that expressed interest in participating, parents or guardians received verbal information about the research. They all gave their written consent to participate in this study voluntarily, and the swimmers agreed before participating.

Participants were reminded that the questionnaires were anonymous and voluntary, and they were encouraged to answer honestly. Swimmers completed the printed questionnaires at the different clubs during a 20–30 min interval. The questionnaires were administered simultaneously to all the members of the club who participated in the research, and an investigator was present to resolve any possible doubts during the administration of the instruments.

### 2.4. Statistical Analysis

**Figure 1:**
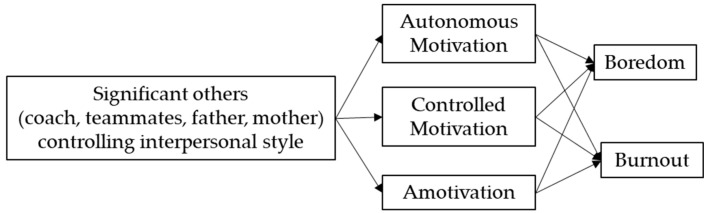
Hypothesized path model of the relationship between significant others’ controlling interpersonal style, forms of motivation, boredom, and burnout.

Descriptive statistics, Pearson correlations, and Cronbach alpha coefficients were analyzed with IBM SPSS Statistics, version 20 (IBM Corp., Armonk, NY, USA). We use path analysis to determine whether there are any meaningful relationships between the variables of the study. Due to the number of parameters in the proposed model, mean scores were employed as indicators of the targeted variables, and two path analysis models were tested using Mplus version 8.1 [54]. One path model considered the sport context (coach and teammates), and the other path model considered the family context (father and mother), as an antecedent of motivation, boredom, and burnout (see Figure 1). The arrows in the figure represent the regression relationships between all the variables in the study. To determine the fit of the models, we considered different indices of fit that included chi-square (χ^2^), the non-normative fit index (NNFI), the comparative fit index (CFI), and the root mean square error of approximation (RMSEA). Values of CFI and NNFI above 0.90 indicate an acceptable fit [55]. For RMSEA, values between 0.05 and 0.10 are considered acceptable, and values equal to or below 0.08 are optimal [56].

## 3. Results

Table 1 presents the descriptive statistics and internal reliability coefficients (Cronbach’s alpha) of the scales used in the study. The mean scores of the swimmers were lower than the mid-point of the scales, except for the variable autonomous motivation. Specifically, mean scores indicated that swimmers perceived that their four significant others (coach, peer, father, and mother) exhibited a low controlling interpersonal style. On average, the swimmers indicated that they experienced autonomous motivation for swimming and got bored very little while practicing their sport. When comparing mean scores, perceptions of the coach’s controlling style were higher (*p* < 0.001) than the perceived controlling style of their teammates (t = 10.17), father (t = 5.03), and mother (t = 5.05). The differences between the perceptions of the mother’s and father’s controlling style were not statistically significant (t = 0.66, *p* = 0.51). Perceptions of the father’s (t = 6.10) and mother’s (t = 5.01) controlling style were significantly higher (*p* < 0.001) than those of teammates.

The reliability coefficients of all the scales were satisfactory (between 0.70 and 0.90), except for the scale of Coach Controlling Style, whose reliability coefficient was marginal (α = 0.66). The correlation between the two items that make up the Boredom scale was 0.30.

Correlation analysis showed that the variables correlate significantly in the expected direction (see Table 1). Overall, a controlling interpersonal style (coach, teammates, father, and mother) was significantly and positively correlated with controlled motivation, amotivation, and burnout. Additionally, coach and peer controlling interpersonal styles were positively correlated with boredom. Associations between controlling style variables and autonomous motivation were not significantly correlated. As Table 1 shows, the strongest correlation is between the controlling styles of the father and the mother, as well as between amotivation and burnout.

**Figure 2:**
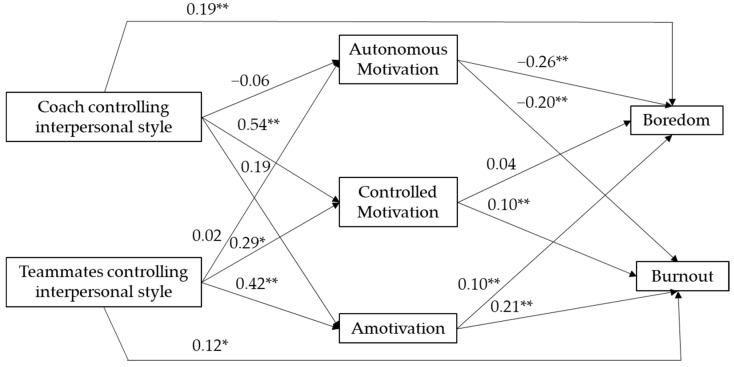
Unstandardized solution of the relationship between coaches’ and teammates’ controlling interpersonal style, forms of motivation, boredom, and burnout. * *p* < 0.05; ** *p* < 0.01.

The hypothesized model that considered the sport context presented an acceptable fit to the data (χ^2^ (5) = 20.09, *p* = 0.001; RMSEA = 0.106; TLI = 0.849; CFI = 0.962; SRMR = 0.043). The modification index indicated adding a path from coaches’ controlling style to boredom and from teammates’ controlling style to burnout. Results of the re-tested model presented a satisfactory fit to the data (χ^2^ (3) = 5.508, *p* = 0.138; RMSEA = 0.056; TLI = 0.958; CFI = 0.994; SRMR = 0.024). The parameters of the standardized solution are displayed in Figure 2. The swimmers’ perceptions of a coach’s controlling style significantly and positively predicted controlled motivation and boredom. Results also indicated that the swimmers’ perceptions of teammates’ controlling style significantly and positively predicted controlled motivation, amotivation, and burnout. Autonomous motivation negatively predicted boredom and burnout, whereas controlled motivation positively predicted burnout, and amotivation positively predicted boredom and burnout. The indirect effect (IE) of coaches’ controlling style on burnout via controlled motivation was significant (IE = 0.05, CI 95% = 0.014–0.091), and the indirect effect of teammates’ controlling style on boredom and burnout via amotivation was significant (IE = 0.05, CI 95% = 0.003–0.082 and IE = 0.05, CI 95% = 0.020–0.156, respectively). Results of the proposed model significantly predicted 27% of the variance in reported boredom and 45% of the variance in reported burnout.

**Figure 3:**
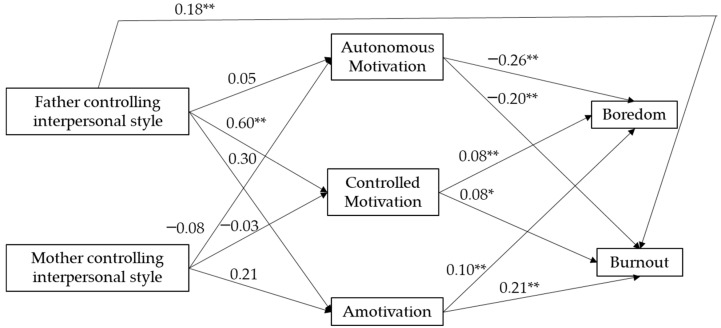
Unstandardized solution of the relationship between parents’ controlling interpersonal style, forms of motivation, boredom, and burnout. * *p* < 0.05; ** *p* < 0.01.

The hypothesized model that considered the family context presented an acceptable fit to the data (χ^2^ (5) = 20.02, *p* = 0.001; RMSEA = 0.106; TLI = 0.846; CFI = 0.961; SRMR = 0.039). The modification index recommended adding a path from fathers’ controlling style to burnout. Results of the re-tested model presented an adequate fit to the data (χ^2^ (4) = 4.90, *p* = 0.297; RMSEA = 0.029; TLI = 0.988; CFI = 0.998; SRMR = 0.024). The parameters of the standardized solution are displayed in Figure 3. The swimmers’ perceptions of their fathers’ controlling style significantly and positively predicted controlled motivation and burnout. Results also indicated that the swimmer’ perceptions of their mothers’ controlling style did not predict any of the forms of motivation. Autonomous motivation negatively predicted boredom and burnout, whereas controlled motivation and amotivation positively predicted boredom and burnout. The indirect effect of fathers’ controlling style on boredom via controlled motivation was significant (IE = 0.05, CI 95% = 0.001–0.098). Results of the proposed model significantly predicted 24% of the variance in reported boredom and 47% of the variance in reported burnout.

## 4. Discussion

The aim of the present study was to explore the mechanisms operating in the relationships between controlling styles of significant others (i.e., coaches, parents, and teammates) and burnout and boredom, and the mediational role of motivation, in a sample of competitive young swimmers. To this end, diverse roles of two contexts of social interaction were assessed: team (i.e., coach and teammates) and family (i.e., father and mother or equivalent social agents).

In relation to the controlling style and its consequences, coaches have been the most studied significant other. Additionally, few studies have examined the parents’ role in their adolescent children’s burnout [10]. As far as we know, there is scarce literature on teammates’ controlling style and its implications for motivation and the quality of the experience of competitive swimming. In our opinion, the lack of studies on teammates’ controlling style shows a gap in the literature because adolescents build their identity, attitudes, values, and feelings from their interactions with teammates, contrasting their issues with them [26]. Hence, teammates’ controlling style may act as an antecedent of maladaptive experiences in athletic participation (e.g., burnout and boredom). In this study, we included teammates and the other three most commonly studied significant others.

As preliminary considerations, we would like to point out that the sample studied perceives interpersonal styles that are not very controlling, with the coaches obtaining the highest scores and only offering moderate values. Likewise, the motivation of these swimmers is clearly self-determined. In line with previous studies (e.g., [29]), the levels of burnout they report are moderate-low.

We analyzed both the family context (mother and father) and the swimming practice context (coach and teammates). All the significant others studied present positive correlations in terms of their controlling styles. In other words, the two significant others that interact with the swimmer in each context had positive associations. Thus, in the family context, we found that when one of the parents has a controlling style, the other tends to have a controlling style as well. These results are in line with Alvarez et al. [3], who found that the interpersonal style of the father and mother presented a high correlation. As suggested by Guay et al. [57], these high correlations would reflect a behavior contagion process, and based on the social learning theory framework [58], parents are mutually influenced and tend to converge, especially in the period of having adolescent children. This relationship is more moderate between coaches’ and teammates’ interpersonal styles. Another contextual motivational construct, motivational climates, has been suggested to be fairly independent from a motivational-created climate when comparing coaches and teammates [59].

In relation to the two contexts of social influence studied, team and family, results have shown a positive association between the controlling style and controlled motivation and amotivation, but not for all the significant others. Thus, on the one hand, coaches’ controlling style directly promotes controlled motivation and boredom in the swimmers. Additionally, coaches’ controlling style indirectly promotes burnout through the mediational role of the controlled motivation of the swimmers. On the other hand, teammates’ controlling style was directly associated with controlled motivation, amotivation, and burnout, and indirectly with boredom and burnout through the mediational role of amotivation. These results are interesting because boredom is related to repetition and lack of variability in the practice, which is a coach’s responsibility (e.g., [38]). Through manipulative mechanisms, controlling coaches force athletes toward behaviors that are not freely chosen. These coaches systematically disregard athletes’ opinions, values, and thoughts, fostering a lack of personal endorsement [60] and interest, which is boredom [36]. In contrast, teammates are more related to sharing feelings and developing attitudes and values. Hence, it seems to make sense that burnout resulting from the subjective assessment of the person’s experience was associated with teammates in a direct way, compared to the coach’s role, which was indirect. As a contextual antecedent of burnout, Ntoumanis et al. [61] studied and compared the effects of coach- and teammate-created motivational climate, showing that the motivational climate created by teammates uniquely contributed to burnout, compared to coach-created climate.

In relation to the family context, only the fathers’ controlling style showed direct associations with controlled motivation and burnout. Additionally, fathers’ controlling style indirectly promotes boredom through the mediational role of swimmers’ controlled motivation. Mothers’ controlling style did not have any significant associations with any of the variables studied. These findings are aligned with previous literature, which pointed out that, when comparing the associations between fathers’ and mothers’ controlling behaviors and their adolescent children’s burnout in sport, fathers had a more important role [10]. Moreover, our results are interesting and complementary to previous studies in the Mediterranean context (and culture). For example, Alvarez et al. [3] showed the main and differential role of the mother compared to the father in the prevention of burnout in their adolescent children when autonomy support was assessed, suggesting that the father’s effect was neutralized by the mother’s autonomy support, that is, the father’s effect on his child’s burnout disappears when the mother uses the autonomy-supportive interpersonal style in her relationship with her child. However, in the present study, when the controlling style is assessed, it is the father who neutralizes the possible effect of the mother’s controlling style, suggesting different roles for fathers and mothers in their children’s emotions.

The present findings provide additional evidence that significant others (coaches, teammates, and parents) are important in the swimmers’ motivation associated with the experience of feeling controlled and pressured in the sport domain. This negative experience promotes controlled motivation and even a lack of motivation, which in turn produce negative outcomes, such as feelings of boredom during the sport practice and higher levels of burnout.

Therefore, the implications of this research are that diverse significant others have different impacts on motivation, burnout, and boredom in adolescent swimmers. It is important to explain to coaches and parents that they play an important role in preventing swimmers’ boredom and burnout. Our results confirm that the use of a controlling interpersonal style (i.e., a controlling use of rewards, using negative conditional rewards, intimidating athletes, exerting personal control over the athletes, and using evaluation and feedback to devaluate athletes) will promote their controlled motivation, burnout experiences, and feelings of boredom, thus increasing the possibility of dropout (e.g., [62]).

This study has some limitations due to its cross-sectional nature and use of self-reported measures. However, these limitations suggest possible directions for future research. Thus, it would be interesting to use measures obtained from direct observation of the environments created in the sports context, extending the study with samples from different sports, as well as longitudinal data collection, which could strengthen the conclusions of the present research about the predictive relationships between the variables studied.

## 5. Conclusions

The results obtained emphasize the negative effect of the controlling interpersonal style of significant others (using coercive strategies, such as the controlling use of rewards, negative conditional attention, intimidation, or excessive personal control) on the motivation of young swimmers, and the development of two indicators of ill-being, boredom during practice, and feelings of burnout. They lead us to reflect on the importance of the environments created by significant others and the adverse effects that their way of interacting and behaving with young swimmers can have.

## 6. Practical Implications

This study highlights the importance of avoiding controlling environments. To this end, educational interventions must be addressed to coaches and parents to prevent athlete burnout and boredom. These programs should have an educational orientation (e.g., [63]). Examples of these programs are interventions in competitive teams (e.g., [64]) and in recreational sport (e.g., [65,66]). The aim should be to create environments (i.e., team and family) where autonomy is supported and controlling behaviors are avoided, both during sport practice and at home, to promote a higher quality sport experience and greater well-being in athletes [67].

## Figures and Tables

**Table 1:** Descriptive statistics, reliabilities, and correlations between study variables.

Variables	Range	*M*	*SD*	Alpha	1	2	3	4	5	6	7	8
1. Coach controlling style	1–5	2.43	0.67	0.66	-							
2. Teammates controlling style	1–5	1.98	0.61	0.70	0.35 **	-						
3. Father controlling style	1–5	2.22	0.71	0.74	0.49 **	0.53 **	-					
4. Mother controlling style	1–5	2.20	0.76	0.79	0.46 **	0.45 **	0.82 **	-				
5. Autonomous motivation	1–7	6.04	0.84	0.89	−0.05	−0.00	−0.01	−0.03	-			
6. Controlled motivation	1–7	2.43	1.20	0.84	0.35 **	0.25 **	0.34 **	0.27 **	−0.10	-		
7. Amotivation	1–7	2.54	1.51	0.85	0.14 *	0.20 **	0.23 **	0.22 **	−0.34 **	0.50 **	-	
8. Boredom	1–4	1.96	0.69	0.30 ^a^	0.26 **	0.15 *	0.12	0.10	−0.40 **	0.28 **	0.39 **	-
9. Burnout	1–5	2.15	0.71	0.90	0.24 **	0.23 **	0.33 **	0.26 **	−0.40 **	0.43 **	0.63 **	0.42 **

^a^ Boredom was assessed by two items, and so the Pearson correlation is reported. * *p* < 0.05, ** *p* < 0.01.

## Data Availability

Not applicable.
